# Biofilm Formation on Hybrid, Resin-Based CAD/CAM Materials for Indirect Restorations: A Comprehensive Review

**DOI:** 10.3390/ma17071474

**Published:** 2024-03-23

**Authors:** Konstantinos Tzimas, Christos Rahiotis, Eftychia Pappa

**Affiliations:** Department of Operative Dentistry, National and Kapodistrian University of Athens, 11527 Athens, Greece; kwstastzimas@dent.uoa.gr (K.T.); effiepappa@dent.uoa.gr (E.P.)

**Keywords:** CAD/CAM, biofilm, resin-based biomaterials, dental materials

## Abstract

Hybrid materials are a recent addition in the field of restorative dentistry for computer-aided design/computer-aided manufacturing (CAD/CAM) indirect restorations. The long-term clinical success of modern dental restorative materials is influenced by multiple factors. Among the characteristics affecting the longevity of a restoration, the mechanical properties and physicοchemical interactions are of utmost importance. While numerous researchers constantly evaluate mechanical properties, the biological background of resin-based CAD/CAM biomaterials is scarcely investigated and, therefore, less described in the literature. This review aims to analyze biofilm formation on the surfaces of novel, hybrid, resin-based CAD/CAM materials and evaluate the methodological protocols followed to assess microbial growth. It is demonstrated that the surface structure, the composition and the finishing and polishing procedures on the surface of a dental restorative material influence initial bacterial adhesion; however, most studies focus on in vitro protocols, and in vivo and/or in situ research of microbiomics in CAD/CAM restorative materials is lacking, obstructing an accurate understanding of the bioadhesion phenomenon in the oral cavity.

## 1. Introduction

Significant advances in the field of restorative dentistry have led to the transition from older metallic dental materials for direct restorations, such as the dental amalgam, to more esthetic, tooth-colored and “tooth-friendly” counterparts, namely composite resin materials. The polymerization process is the critical drawback concerning using these restorative materials for direct intraoral applications. Residual monomers and polymerization shrinkage reduce their clinical success [1]. Further disadvantages of direct resin-based restorations include inferior mechanical strengths, rapid occlusal and proximal wear, marginal discoloration, loss of integrity, a low fracture toughness and postoperative sensitivity [2]. The limitations of this direct, sensitive approach have been partially overcome by the development of nano-filled and nano-hybrid direct composite resins and by the application of indirect laboratory methods [3,4,5,6].

Furthermore, indirect restorations, either by using resin-based materials or ceramics, have proven to be a viable alternative therapeutic modality [7]. Due to the fact that ceramics have long been characterized as expensive, brittle materials that induce wear to the opposing dentition and are not repairable after fracture, indirect resin-based restorations are continuously gaining attention [8,9]. The everlasting need for more conservative, minimally invasive, and, at the same time, predictable procedures that maximize patients’ comfort has led to the incorporation of digital means in the fabrication of dental restorations. The introduction of computer-aided design/computer-aided manufacturing (CAD/CAM) appliances followed the rising demand for digital dentistry, and, subsequently, the dental market was overrun with new dental biomaterials for several types of restorations (inlays, onlays, endocrowns, etc.) [10,11,12]. The first subtractive manufacturing materials used were feldspar ceramic blocks [13]. Although they are strong, ceramics are brittle materials with a low fracture toughness and a high susceptibility to failure in the presence of flaws [9]. Therefore, using “hybrid ceramic” or resin-based CAD/CAM restorative materials has proven an ideal alternative. Their main benefit is based on adequate factory polymerization, involving high-heat and high-pressure techniques, eliminating polymerization defects and monomer release in this way. Simultaneously, incorporating a more significant amount of filler particles and altering the polymer matrix enhance their mechanical properties. The hybridity of these newly introduced CAD/CAM blocks depends on the common goal of combining the positive effects of ceramic and resin-based components [14]. Since the flexural strength of hybrid resin-based CAD/CAM blocks is higher than that of recently developed nano-filled composite resins and their elastic modulus is similar to that of dentin, a more uniform stress distribution during loading may be anticipated [15].

**Figure 1:**
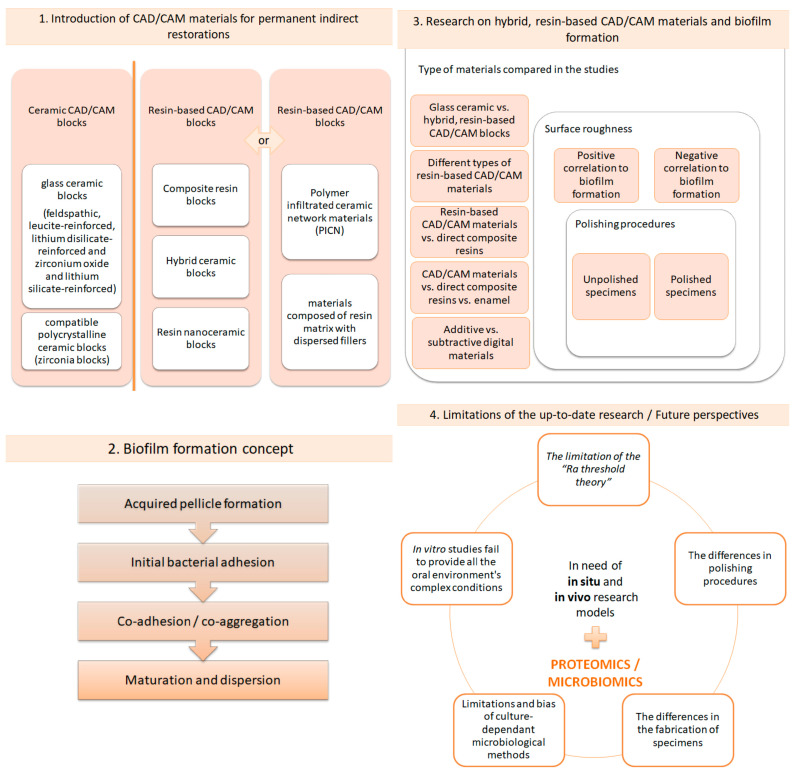
Succinct description of the sections of this review.

Through the years, researchers have constantly evaluated the mechanical properties of hybrid, ceramic, resin-based CAD/CAM blocks. The flexural strength, Vickers hardness and elastic modulus are of utmost importance for excellent clinical performance. Surface properties, such as the surface roughness and surface topography, have also been investigated, but to a lesser extent compared to mechanical properties [15,16,17,18,19,20,21,22,23,24,25]. Unfortunately, scarce evidence exists concerning bacterial attachment and subsequent biofilm formation on hybrid ceramic, resin-based CAD/CAM blocks for permanent, indirect restorations, meaning that this is a field that needs further investigation. Biofilm formation is a potential causative factor facilitating restoration failure since it promotes the appearance of secondary caries on the restoration’s margins and provokes biodegradation, thus altering the restorative material’s surface characteristics [26,27]. To the best of our knowledge, until recently, no critical reviews of the existing literature focusing on bacterial formation on CAD/CAM dental materials for indirect restorations have been published, and a comprehensive evaluation of the methodology, observations and results of current research protocols is lacking. Therefore, the aims of this review are, firstly, to introduce the resin-based CAD/CAM materials used for single indirect restorations and to present the recent data concerning biofilm formation on their surfaces, and secondly, to shed light on the methodological patterns used, as well as their limitations. Furthermore, future directions in microbiome analysis will be highlighted. A visualization of the structure of this comprehensive review is presented in Figure 1.

## 2. “Hybrid”, Resin-Based Materials in the Digital Dentistry Era

There are a lot of different classification systems regarding CAD/CAM blocks and their application in contemporary restorative dentistry. The raw classes of CAD/CAM blocks fabricated for single, permanent indirect restorations are *ceramic CAD/CAM blocks* and *resin-based CAD CAM blocks*. According to their composition and microstructure, ceramic CAD/CAM blocks can be further divided into *glass ceramics*, subcategorized into feldspathic, leucite-reinforced, lithium-disilicate-reinforced and zirconium-oxide- and lithium-silicate-reinforced ceramic blocks, and *compatible polycrystalline ceramics*, namely zirconia CAD/CAM blocks [12]. The CAD/CAM blocks that incorporate a resin-based organic matrix can be subcategorized as follows: *polymer-infiltrated ceramic network materials* and *materials composed of a resin matrix with dispersed fillers* [28,29]. Other resin-based CAD/CAM block classes include *composite resin* CAD/CAM blocks, *hybrid ceramic* CAD/CAM blocks and *resin nanoceramic* CAD/CAM blocks [12,14,30]. The latter refers to polymeric networks that are reinforced with ceramic fillers (ceramics, glass ceramics, glasses, ultrafine glass particles, nanohybrid fillers, etc.). The term “hybrid” is often misinterpreted and should only be used to describe a CAD/CAM block that consists of a polymer-infiltrated ceramic network (PICN). This CAD/CAM block (VitaEnamic, VITA Zahnfabrik, Bad Säckingen, Germany) presents a double network hybrid structure composed of a porous, pre-sintered ceramic network, conditioned by a coupling agent and infiltrated with a polymer via capillary action [31,32,33]. Caution is required, since the misclassification of CAD/CAM materials in the dental literature is significant and might lead to misuse and incorrect clinical identification of CAD/CAM materials [34]. Although resin-based, hybrid ceramic, nanoceramic CAD/CAM materials exhibit inferior optical properties, their advantages compared to traditional glass ceramics are summarized as follows: they are not stiff, brittle materials; they mimic the structure of natural tooth components; they present direct composite repairability; and they are more easily and quickly fabricated [9]. Moreover, resin-based materials may be less susceptible to chipping during the milling procedure [35]. Occlusal and proximal adjustments (polishing procedures) are much more easily accomplished [14,36].

The most used resin-based CAD/CAM blocks are summarized in Table 1.

According to the manufacturer, the polymer-infiltrated ceramic network material (Vita Enamic, VITA Zahnfabrik, Bad Säckingen, Germany) consists of 86% filler by weight and 14% UDMA and TEGDMA polymer network by weight. More precisely, the inorganic fillers are primarily silicon dioxide and aluminum oxide and secondarily sodium, potassium, and calcium oxide, as well as boron trioxide and zirconia [37,38,39]. One commonly used resin nanoceramic CAD/CAM material is Lava Ultimate (3M ESPE, St. Paul, MN, USA). Nanomers of 20 nm in diameter made from silica and 4–11 nm in diameter made from zirconia, as well as zirconia and silica nanoclusters of 0.6–10 μm, comprise the approximately 80% by weight inorganic filler content, which is placed in an organic matrix of Bis-GMA, UDMA, Bis-EMA, and TEGDMA [28,40]. Shofu Block HC (Shofu Inc., Kyoto, Japan) is described as a ceramic-based restorative material, consisting of 61% silica powder, zirconium silicate and microfumed silica in a UDMA and TEGDMA organic matrix [28,41]. Cerasmart (GC Corporation, Tokyo, Japan) is now off the market and has been replaced by Cerasmart 270, which is described as a force-absorbing hybrid ceramic CAD/CAM block. Its predecessor’s composition included Bis-MEPP, UDMA, DMA, silica (20 nm) and barium glass (300 nm). Its inorganic filler load was 71% by weight [28,42]. Grandio Block (VOCO GmbH, Cuxhaven, Germany) is described as a nano-hybrid CAD/CAM block of 86% by weight nanoceramic filler particles in a UDMA and DMA organic matrix [43]. Another often used resin-based material is Brilliant Crios (Coltene Whaledent AG, Altstätten, Switzerland), described by the manufacturer as a reinforced composite block for permanent restorations. It consists of a cross-linked methacrylate resin matrix (Cross-Bis-GMA, Bis-EMA and TEGDMA) and 70.7% by weight dental glass (barium glass < 1.0 μm) and amorphous silica (<20 nm) [44]. Katana Avencia Block (Kuraray Noritake Dental, Tokyo, Japan) consists of UDMA, other methacrylate monomers and mixed fillers of colloidal silica and aluminum oxide and was launched as a hybrid, ceramic, composite resin CAD/CAM block [45]. Lastly, Tetric CAD (Ivoclar Vivadent AG, Schaan, Lichtenstein) is composed of cross-linked methacrylates, such as Bis-GMA, Bis-EMA, TEGDMA and UDMA, and 71% by weight barium glass (<1 µm) and silicon dioxide fillers [46]. As observed, resin-based CAD/CAM materials have almost the same microstructures, but in different proportions.

## 3. The Concept of Biofilm Formation

The oral microbiome, hosting approximately 700 different species of bacteria, represents the second largest microbiota environment, following the gut microbiome [47]. The oral cavity is a complex host with unique anatomical structures, including hard (natural teeth and restorative materials) and soft tissues (oral mucosa). The oral microbiome is the sum of the oral microbes, their genetic information, and the oral environment in which all components interact [48]. The so-called “climax community”, consisting of dietary habits, environmental conditions, host genetics and early microbial exposure, plays a pivotal role in the oral microbiota composition [49]. Biofilms are formed on every existing surface (soft and hard tissues, dental materials, etc.) in the oral cavity. The presence of biofilms is not necessarily malicious per se, since under normal circumstances, pathogenic and physiological microorganisms exhibit a phenomenon called symbiosis, which leads to the maintenance of oral health [50]. Several factors may disrupt this sensitive balance and result in dysbiosis (imbalance of the microbiome). Inadequate oral health conditions and dietary habits rich in low-molecular-weight carbohydrates, as well as inflammatory and autoimmune disorders, create the ideal environment for the establishment of pathological processes, such as the demineralization of tooth structures, tooth decay, secondary caries at the margins of restorative materials, gingivitis–periodontitis–peri-implantitis, tooth loss and/or stomatitis [51,52]. Biofilm formation (dental plaque) is a multiple-stage process [53]. When a dental biomaterial, in our case a resin-based CAD/CAM material, is adhered to a tooth structure and starts functioning in the oral cavity, it is immediately coated by saliva, and an acquired pellicle is formed [54]. After the first stage of acquired pellicle formation, the initial bacterial adhesion commences, and the formation of the dental plaque biofilm continues with the adhesion and coagulation of further microorganisms. Maturation, followed by dispersion, leads to the final dental plaque composition [55]. More precisely, the acquired pellicle is a noncellular, micellar structure that is composed of salivary glycoproteins, phosphoproteins, lipids and components of gingival crevice fluids, plus microbial products (glycosyltrasferases and glycans). The acquired pellicle modifies the surface properties of the dental biomaterial and alters the interactions between the biomaterial and the host response [56,57]. The salivary molecules activate receptors, which interact with adhesins on the surfaces of bacteria [58]. The bacterial conjunction is divided into three categories, depending on the distance between bacteria and the dental surface. If the distance is greater than 100nm, the initial bacteria are transported to the point of interest via natural salivary flow, Brownian motion (fluid dynamics) and chemotaxis (chemical signaling).

**Figure 2:**
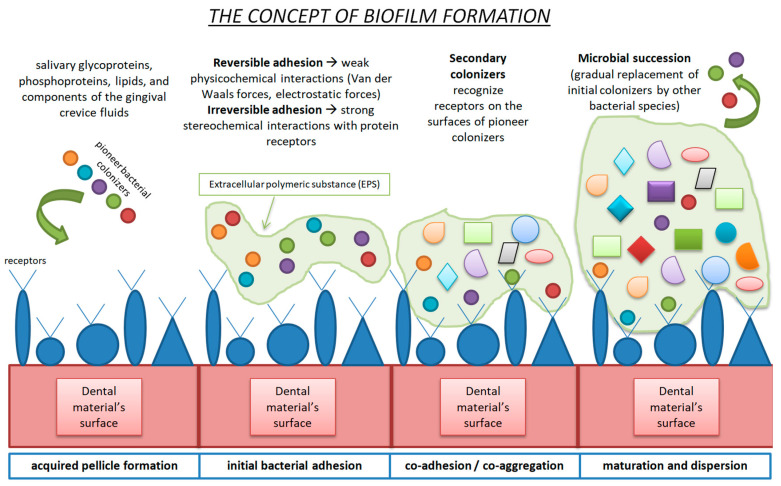
Schematic representation of biofilm formation.

When the distance between the bacteria and the surface is 20 to 100 nm, van der Waals forces and electrostatic interactions are of utmost importance for cell attachment. Lastly, when the distance is short (<20 nm), biofilm attachment due to nonspecific and specific bonding mechanisms is observed. Signaling transactions, as well as activation of specific transmembrane receptors, are examples of specific bonding mechanisms. After the arrival of microorganisms, bacterial attachment commences and pioneer colonizers are established [59]. The initial binding is reversible due to the weak physicochemical interactions (van der Waals and electrostatic forces). The next step is the irreversible phase, where strong stereochemical interactions between microbial adhesins and receptors on the acquired pellicle occur. Adhesins expressed by secondary colonizers recognize receptors on the surfaces of pioneer colonizers, and the co-aggregation or co-adhesion phase takes place. Microbial succession, meaning the gradual replacement of initial colonizers by other bacterial species through the initial bacteria’s metabolic process, follows, and mature dental plaque is built [49,60]. Figure 2 briefly describes the dental plaque formation stages.

All in all, bacterial colonization, especially at its early stage, is contingent upon detachment shear forces and the surface energetic state of the substrate. The decisive role of surface roughness, surface free energy, surface wettability, surface topography and surface chemical composition on biofilm formation is scientifically documented, mainly by in vitro studies [61,62,63,64]. An increased surface roughness promotes greater bacterial attachment due to the greater surface contact area available for adhesion, the presence of stagnation points and the shielding of microbial cells from shear forces. Bacteria adhere easily to a surface with a high surface energy (hydrophilic), rather than to a substrate with a low surface energy [65,66]. However, since a plethora of factors has been proven to be responsible for the alterations at the interface between the substratum and biofilms, a cautious interpretation of the literature and further investigations into the correlation of surface characteristics and biofilm formation are necessary. Furthermore, it should not be forgotten that the properties of a dental material have a significant effect on the biofilm and that the biofilm may conversely affect and alter the material properties [67,68].

## 4. Research on Biofilm Formation on Resin-Based, Hybrid CAD/CAM Materials

Research focusing on biofilm formation on resin-based CAD/CAM materials for permanent indirect restorations predominantly originates from in vitro studies. An overall overview demonstrates a possible correlation between biofilm formation and surface characteristics (mainly surface roughness), as well as a strong association between bacterial growth, surface roughness and surface modification techniques (polishing procedures).

More precisely, after a thorough investigation of the recent literature concerning biofilm formation on resin-based CAD/CAM blocks for permanent indirect restorations, a total of eleven research articles were found [69,70,71,72,73,74,75,76,77,78,79]. These studies investigated one or more hybrid, resin-based CAD/ CAM materials with regard to biofilm attachment and growth. They evaluated either the biofilm formation as an independent variable or biofilm formation in association with surface characteristics, such as surface roughness and surface free energy. The materials investigated in each study differed. Some researchers solely examined resin-based CAD/CAM blocks [70,76,77,78]. Others compared resin-based CAD/CAM blocks to conventional composite resins [74], whereas some in vitro research incorporated direct composite resins, indirect CAD/CAM blocks and human enamel [72,73]. Moreover, other studies focused on ceramic CAD/CAM materials and hybrid resin-based CAD/CAM materials [69,71]. Lastly, a newly conducted in vitro study compared CAD/CAM-manufactured resin-based materials for indirect restorations with 3D-printed resin-based materials [79]. Other researchers investigated the potential correlation between the surface modification procedures on CAD/CAM resin-based materials and increased or decreased biofilm formation. In this kind of research, control groups were not subjected to further surface treatments, in contrast to the experimental groups, where finishing and polishing procedures with specific grinding and polishing protocols established by each researcher took place. Most in vitro studies used *Streptococcus mutans* (*S. mutans*) as the monospecies for bacterial adherence to the tested materials. Other bacterial strains used were *Candida albicans* (*C. albicans*), *Streptococcus sanguis* (*S. sanguis*), *Streptococcus gordonii* (*S. gordonii*) and *Lactobacillus* species. Only two in situ studies, which tried to identify the biofilm formed on smooth restorative materials, integrated hybrid resin-based CAD/CAM materials into their experimental groups [72,75].

The methods used for the evaluation of surface properties and the assessment of biofilm formation are scientifically documented by former researchers. Using a stylus profilometer or a 3D optical profilometer in contact or non-contact mode is the gold standard in the assessment of surface roughness [27,80]. Most researchers measuring surface roughness record and compare the Sa value (arithmetical mean height, expressing, as an absolute value, the difference in height of each point compared to the arithmetical mean of the surface). Scanning electron microscopy (SEM) provides qualitative information on the surface structure of a dental material [81]. Furthermore, the use of attenuated total reflectance, Fourier transform infrared spectrometry (ATR–FT–IR spectrometry) and energy-dispersive X-ray microanalysis (EDX microanalysis) enriches protocols with information concerning the molecular composition and elemental analysis of the surfaces tested (surface topography and chemical composition assessment) [82,83,84]. The sessile drop method calculates the surface free energy using contact angle measurements and customized optical goniometers [85]. For the microbiological analysis of the tested specimens, various diverse methods (direct as well as indirect) have been introduced. Still, the most commonly used method is the application of a bioreactor followed by colony-forming unit counting (CFU/mL). Scanning electron microscopy (SEM) and confocal scanning laser microscopy (CSLM) are supplementary qualitative methods for biofilm evaluation [86].

The objectives, the experimental methods and the results of these studies are analyzed on a large scale in Table 2.

## 5. Limitations of the Current Research

Delving deeper into the aforementioned research, a cautious interpretation of their ambiguous results should be accomplished.

On the one hand, when evaluating resin-based CAD/CAM materials, a group of researchers demonstrate a definite association between biofilm formation and surface roughness or surface modification procedures [69,70,71,74,75,77,78], whereas, on the other hand, no correlation between these factors is found in research studies conducted by other groups of investigators [73,76,79]. These discrepancies are also present in previously conducted in vitro studies assessing surface roughness, different polishing techniques and their impact on biofilm formation for laboratory-fabricated indirect and direct resin-based restorative materials [27,87,88,89,90,91,92,93,94,95,96].

This divergence may rely on the following factors:

1. The Ra threshold theory of 0.2 μm.

In several studies that incorporate CAD/CAM samples in their protocols, with initial Sa values of samples greater than 0.2 μm, a positive correlation between surface roughness and bacterial attachment has been found [69,70,77]. Additionally, it is further demonstrated that surface roughness has an insignificant effect on bacterial adhesion when the Sa values of the tested specimens are below this threshold [97]. In the research protocol of Ionescu et al. in 2020, where surface roughness values (Sa) were less than 0.2 μm, no strong correlation between Sa and bacterial adhesion was present [73]. Interestingly, in some research protocols with Sa values greater than the 0.2 μm threshold, no correlation between the two investigated factors has been observed [76,79], and in other research where the Sa values were lower than the established threshold, a strong correlation between surface roughness and biofilm adhesion has been demonstrated [74,78]. This fact highlights the potential influence of additional factors, such as polishing procedures, chemical composition and topography, on the outcomes of bacterial adhesion. Moreover, a systematic review by Duetra et al. in 2018 [98] concluded that the impact of roughness on bacterial adhesion is not related to a roughness threshold but rather to a range of surface roughness, which is wide and material-dependent. The majority of in vitro studies evaluating either the surface roughness as a single parameter or the relationship between surface roughness and bacterial colonization use only the Sa value, which is a single height parameter of a surface. Additional spatial, functional or hybrid (e.g., developed interfacial area ratio, Sdr) parameters, may give a greater insight into surface texture and bacterial colonization.

2. The polishing procedure may affect bacterial adhesion on resin-based CAD/CAM materials for indirect restorations.

CAD/CAM materials directly after their milling procedure present an insufficient smoothness, which may be adjusted by additional polishing protocols [99]. Although no standard protocol for polishing CAD/CAM restorations has been established [100], each company manufacturing CAD/CAM resin-based materials fabricates and promotes its finishing and polishing sets to achieve optimal surface characteristics in the final restoration. According to the literature, finishing and polishing protocols affect the surface roughness of dental materials and promote a heterogeneous impact on bacterial adhesion [98]. Comparing polished resin-based CAD/CAM blocks to unpolished control groups, statistically significant differences were found concerning the decreased amount of bacterial adhesion on polished specimens [70,71,74,78]. It is evident that different polishing techniques remove the superficial layers of the tested materials, resulting in a physically as well as chemically altered surface compared to the unpolished control group and in a subsequently reduced surface roughness [101,102]. Meanwhile, significant differences in surface roughness values were obtained while using the same polishing protocols for different resin-based CAD/CAM materials. This may be attributed to the third factor that generates variance in the results of the studies mentioned above, namely the elemental composition and the microstructure of resin-based CAD/CAM materials.

3. The chemical and topographical microstructure of hybrid, resin-based CAD/CAM materials.

More precisely, a different structural composition is present in lithium disilicate glass ceramic CAD/CAM blocks compared to polymer-infiltrated ceramic network materials, nano-ceramic filler-infiltrated polymer networks or direct resin-based materials, leading subsequently to different surface roughness and biofilm adherence values. Furthermore, biofilm formation is positively linked to the amount of the resin matrix rather than the amount of filler particles. It is scientifically proven that some released monomers stimulate bacterial growth [90]. This may explain the fact that in the research of Hassan et al. in 2022 [76], Brilliant Crios blocks exhibited more outstanding bacterial adhesion compared to Vita Enamic and Cerasmart blocks, since the former contain a greater proportion of resin matrix (29%wt). It should not be forgotten that CAD/CAM blocks are produced under a high pressure and a high temperature, improving their properties. This should be counted as an additional factor explaining the reduced biofilm formation on these materials compared to conventional composite resins [9,19].

All in all, the type of resin-based CAD/CAM material and the surface finishing and polishing techniques are significantly related to surface roughness and biofilm adherence.

4. The lack of standardization in the fabrication of specimens.

The results of the research protocols of Contreras-Guererro et al. in 2020 [74] are opposed to other similar in vitro studies evaluating biofilm formation on ceramic CAD/CAM, hybrid resin-based CAD/CAM and composite resin specimens, since they demonstrate greater surface roughness and biofilm formation values for the hybridized resin-based CAD/CAM blocks compared to conventional composite resins. Kim et al. in 2017 [69] also demonstrated that simulated intraoral adjustment and polishing procedures have a negative effect on surface roughness and on biofilm formation in hybrid resin-based materials, leucite-reinforced glass ceramics and nanoleucite-glass ceramics compared to their unpolished counterparts. Such discrepancies may be justified by the disparities in the preparation of the specimens between different research protocols. For the fabrication of conventional composite resin specimens, a universal approach has been proposed using molds with specific dimensions, glass slides, and acetate strips. On the other hand, for the fabrication of CAD/CAM samples, several approaches have been used. Some researchers generated CAD/CAM samples by the use of a diamond bur or a trepan bur under a constant water flow [73,75], whereas some others used diamond discs attached to low-speed straight handpieces [69]. In two research protocols, CAD/CAM samples were fabricated by the use of a milling unit [72,74]. Most researchers used a low-speed precision cutting machine and a diamond blade under flowing water [70,76,77,78,79]. All these different fabrication methods may result in different study outcomes.

Furthermore, in some studies, finishing and polishing were accomplished by the use of grinding and polishing devices under a constant water flow combined with silicone carbide grinding papers of different grit sizes, and the specimens were additionally polished by polishing sets of different manufacturers, whereas some others used several polishing systems on the fabricated (by the use of rotary instruments) specimens directly. These variations in the methodology of experimental protocols result in divergent outcomes in the research. All we need is the standardization of the procedures and the establishment of ideal conditions that can mimic, to the greatest extent, the intraoral environment. In vitro studies fail to provide all the oral environment’s complex conditions, and future research should focus on in situ and in vivo protocols.

5. The biofilm assessment method

Referring to intraoral conditions, another factor affecting the results of biofilm formation on resin-based CAD/CAM materials is the method of biofilm assessment. Most in vitro studies use one microbial strain (monospecies colony), mainly *S. mutans*, since it is a well-known predominant cariogenic species [79]. A plethora of artificial systems try to mimic the intraoral environmental conditions for biofilm development on the surface of a dental material; these systems are called bioreactors. They are used for in vitro biofilm growth and are categorized either as static or dynamic bioreactors. They can be made of artificial oral microcosms, single species or defined consortia of a few species growing together [103,104]. Most in vitro studies assessing biofilm formation on resin-based CAD/CAM surfaces use a single species, since this is a simple, controlled, inexpensive, highly reproducible technique [105]. Attempting to imitate oral conditions, most in vitro studies incorporate in their microbiological protocol the immersion of samples in mucin containing artificial saliva or whole mouth saliva, secreted from a volunteer, to form the acquired pellicle. Colony-forming unit counting (CFU/mL), combined with SEM investigations and confocal laser scanning microscopy (CLSM), is used to perform qualitative and quantitative evaluations of bacterial formation [106]. SEM and CLSM have limitations, including the high cost and complexity of their protocols, the inability of CLSM to discriminate strains, the inability of SEM to discriminate live and dead bacteria, and the fact that only a specific selected area of the substrate may be evaluated [107].

Furthermore, bacterial adhesion on the surface of a substratum is not only influenced by the surface characteristics of the materials tested but also by the selected bacterial strain, the growth medium used and the specific adhesion mechanisms of the selected monospecies. Only one in vitro study by Ionescu et al. in 2020 [74] used four models of bioreactors for microbial investigations (static, orbital shaking, continuous flow and mixed-plaque formation bioreactors) to assess biofilm formation on resin-based CAD/CAM materials, concluding that, when bioreactors with shear forces or bioreactors where multi plaque formation takes place are used, lower *S. mutans* formation on resin-based CAD/CAM blocks was observed compared to conventional composite resin specimens. Unfortunately, in vitro biofilm formation has only been investigated via culture-dependent, close-ended molecular methods with a great risk of bias which do not coincide with real in vivo conditions.

Until recently, only two in situ studies that evaluated biofilm adhesion and formation on different dental restorative materials used a resin-based CAD/CAM material in their experimental groups [72,75], meaning that this is a field that nowadays attracts the interest of a lot of researchers.

Lastly, it should not be forgotten that under clinical conditions, surfaces are immediately coated by saliva and the composition, the flow and the volume of saliva differ based on neural control system signaling, as well as on physical, environmental and/or pathological factors, which include circadian rhythm, age, gender, physical exercise, oral hygiene, food consumption (diet), medication and systematic diseases [108]. It is almost impossible to mimic all these above-mentioned conditions in in vitro protocols; therefore, in vivo studies incorporating parts of these factors in their study design should be conducted.

## 6. Conclusions

Newly introduced CAD/CAM restorative materials are gaining attention due to their more than satisfactory mechanical properties. The biological background of the tested dental materials proves to be a significant factor in dental science since bacterial adhesion is inextricably linked to secondary caries on the margins of a restoration and subsequently to the good or the poor clinical performance of a restoration. Bacterial adhesion on CAD/CAM resin-based materials is primarily investigated in in vitro studies that, unfortunately, do not represent the exact conditions of the oral environment. The current literature demonstrates a possible interaction between biofilm formation and the surface of the substratum. Surface roughness, surface free energy, surface topography and elemental and chemical composition may have a crucial impact on biofilm growth, mainly in the early stages of bacterial adherence. Further studies should be conducted in order to shed light οn the unknown phenomenon of bioadhesion.

## 7. Future Perspectives

When conducting an in vitro study, caution should be exercised concerning the standardization of the applied procedures. Since in vitro studies present, inter alia, culturing bias, the scientific interest of most researchers focuses on the use of culture-independent methods for the identification of the total bacterial community in the oral environment. To do so, open-ended genome sequencing technologies, such as next-generation sequencers (NGSs), as well as proteomic and metaproteomic techniques that may identify the host and the microbial proteome, are gradually being incorporated in the microbiological armamentarium. The conduction of in situ and/or in vivo studies using resin-based CAD/CAM restorative materials as experimental groups and human enamel and conventional composite resins as control groups, incorporated on oral splints worn by volunteers, may provide an insight into how surface characteristics, saliva, acquired pellicles and the oral microbiome interact. Interestingly, via 16S ribosomal RNA gene sequencing, the whole microbiome present in biofilms may be identified [109]. Furthermore, mass spectrometry (MS) devices may provide information concerning the proteomic profile of a tested material. Utilizing specific databases of bioinformatics, bacterial species adhered to a surface may be recognized using MS (metaproteomics). The “-Omics” era focuses on the principle that the whole organism works in synergy, and each bacterium is dependent on the other species present. Since biofilms are described as conglomerates, a more holistic, ecological approach to controlling dental biofilms is necessary.

## Figures and Tables

**Table 1:** Commonly used hybrid, resin-based CAD/CAM materials in the dental market.

Hybrid, Resin-Based CAD/CAM Material	Description	Manufacturer	Composition
Vita Enamic	Polymer-infiltrated ceramic network material (PICN) Hybrid ceramic block	VITA Zahnfabrik, Bad Säckingen, Germany	86% by weight inorganic fillers (mainly silicon dioxide and aluminum oxide) 14% organic matrix by weight: UDMA and TEGDMA
Lava Ultimate	Resin nanoceramic block	3M ESPE, St. Paul, MN, USA	80% by weight inorganic fillers (nanomers of silica and zirconia and zirconia and silica nanoclusters of 0.6–10 μm) 20% organic matrix: Bis-GMA, UDMA, Bis-EMA and TEGDMA
Shofu Block HC	Hybrid ceramic block	Shofu Inc., Kyoto, Japan	61% inorganic fillers (silica powder, zirconium silicate, and microfumed silica) Organic matrix: UDMA and TEGDMA
Cerasmart	Force-absorbing hybrid ceramic block	GC Corporation, Tokyo, Japan	71% by weight inorganic fillers (silica (20 nm) and barium glass (300 nm)) Organic matrix: Bis-MEPP, UDMA, DMA
Grandio Bloc	Nanoceramic hybrid block	VOCO GmbH, Cuxhaven, Germany	86% by weight inorganic fillers Organic matrix: UDMA and DMA
Brilliant Crios	Reinforced composite block	Coltene Whaledent AG, Altstätten, Switzerland	70.7% by weight inorganic fillers (barium glass and amorphous silica) Organic matrix: Cross-Bis, GMA, Bis-EMA and TEGDMA
Katana Avencia Block	Hybrid ceramic composite resin CAD/CAM block	Kuraray Noritake Dental, Tokyo, Japan	82% by weight inorganic fillers (colloidal silica and aluminum oxide) Organic matrix: UDMA and other methacrylate monomers)
Tetric CAD	Composite block	Ivoclar Vivadent AG, Schaan, Lichtenstein	71% by weight barium glass (<1 µm) and silicon dioxide fillers Organic matrix: cross-linked methacrylates, (Bis-GMA, Bis-EMA, TEGDMA, UDMA)

**Table 2:** Research focusing on bacterial adhesion on hybrid resin-based CAD/CAM materials for indirect restorations.

Objective	Type of Specimens/Type of Control Group	Tests	Conclusion	Study/Year
Evaluation of surface roughness and biofilm formation on CAD/CAM materials before and after polishing	(1) Vita Enamic, Vita Zahnfabrik (2) Lava Ultimate, 3M ESPE (3) Vitablocs Mark II, VITA Zahnfabrik, Bad Säckingen, Germany (4) Wieland Reflex Veneering porcelain, Wieland Dental, Pforzheim, Germany POLISHING PROCEDURES Unpolished specimens (control group) Uniformly polished specimens with diamond burs, finishing burs and extrafine porcelain burs (experimental group)	(1) SEM, CLSM, crystal violet assay for microbial analysis of *S. grodonii* (2) 3D Slicer software for surface roughness evaluation	More irregular surface topography in polished specimens compared to controls Greater surface roughness (*Ra*) values in polished CAD/CAM blocks compared to controls. Greater biofilm growth on polished specimens compared to controls	Kim et al., 2017 [69]
Evaluation of the surface topography and bacterial adhesion CAD/CAM blocks after different surface finishing procedures	(1) Vita Enamic, Vita Zahnfabrik (2) Lava Ultimate, 3M ESPE POLISHING PROCEDURES (1) No surface finish (control group) (2) Diamond bur surface finish (3) Polishing system for hybrid ceramics (4) Polishing system for ceramics	(1) Stylus profilometer for surface roughness evaluation (Ra, Rz, Rq height parameters) (2) Spectrophotometry, CFU/mL, SEM and CSLM for microbial analysis of *S. mutans*	Surface roughness and bacterial adhesion are lower for Vita Enamic compared to Lava Ultimate, regardless of the finishing procedures The type of material and the finishing techniques have an effect on surface roughness and bacterial adhesion	Hammerschnitt et al., 2018 [70]
Comparison of biofilm formation on CAD/CAM materials in accordance with their roughness	(1) Vita Enamic, Vita Zahnfabrik (2) IPS Empress, Ivoclar Vivadent AG, Schaan, Lichtenstein (3) IPS Empress Multi, Ivoclar Vivadent AG, Schaan, Lichtenstein (4) IPS emax, Ivoclar Vivadent AG, Schaan, Lichtenstein, before and after sintering POLISHING PROCEDURES Unpolished specimens (control group) were uniformly polished with 800–1200 grit sandpaper discs (experimental group)	(1) Powder X-ray diffraction pattern (XRPD) and (ATR–FT–IR) for surface topography evaluation (2) Contact angle measurement for wettability evaluation (3) Fluorescence microscopy and CFU/mL counting for microbial analysis of *S. mutans*, *C. albicans* and *Lactobacillus rhamnosus*	Non-polished surfaces are more susceptible to biofilm adhesion compared to their polished counterparts The degree of biofilm formation depends on the tested microbial species	Dobrzynski et al., 2019 [71]
Identification and comparison of the oral microbiome on resin-based materials in vivo and in vitro	(1) Grandio flow, VOCO GmbH, Cuxhaven, Germany (conventional flowable composite resin) (2) Grandio Bloc, Voco GmbH (resin-based CAD/CAM material) (3) Bovine enamel (control group)	For the in situ project: 15 volunteers wore oral splints with slabs of resin-based materials and bovine enamel for 48 h, and Ilumina Miseq Next Generation Sequencing of 16S ribosomal RNA (V1–V2 regions) for bacterial identification followed	No significant differences in bacterial colonization for the different dental composites and the control group in vivo	Conrads et al., 2019 [72]
Differences in biofilm formation between indirect CAD/CAM resin-based composites and their direct resin-based counterparts	(1) Grandio Bloc, VOCO GmbH (2) Lava Ultimate, 3M ESPE (3) Katana Avencia, Kuraray Corp. (4) Vita Enamic, Vita Zahnfabrik (5) Grandio SO, VOCO GmbH, Cuxhaven, Germany (6) Filtek Supreme XTE, 3M ESPE, St. Paul, MN, USA (7) Ionostar Plus, VOCO GmbH, Cuxhaven, Germany (positive control) (8) Human enamel (negative control) POLISHING PROCEDURES All specimens were uniformly finished and polished with silica–alumina grinding papers (600–4000 grit) and stored in artificial saliva	(1) Profilometry in contact mode for surface roughness evaluation (Ra height parameter) (2) SEM/EDX analysis and X-ray diffraction (XRD analysis) for molecular, elemental and structural analysis of the specimens. (3) Thermogravimetric analysis (TG) and differential scanning calorimetry (DSC) for quantification of filler content of the specimens. (4) Static, orbital shaking, continuous flow and mixed- plaque formation bioreactors for microbial investigation of *S. mutans* and mixed plaque biofilm	CAD/CAM blocks yielded lower *S. mutans* and mixed plaque biofilm formation compared to direct resin-based materials No strong correlation between biofilm formation and surface roughness Stronger corellation between biofilm formation, manufacturing techniques and curing processes	Ionescu et al., 2020 [73]
Evaluation of biofilm formation on different dental restorative materials	(1) IPS Emax Press, Ivoclar Vivadent (2) IPS Emax CAD, Ivoclar Vivadent (3) Lava Ultimate, 3M ESPE (4) Vita Enamic, Vita Zahnfabrik (5) Two conventional composite resins POLISHING PROCEDURES CAD/CAM specimens subjected to sandblasting, polished by sandpaper discs (180–2000 grit), Sof–Lex discs, green stone and rubber points. Composite resins polished with polishing brushes, Sof–Lex discs, diamond paste and cotton tassel	(1) Atomic force microscopy for surface roughness evaluation (Ra, Rmax, Rz height parameters) (2) Dynamic bioreactor, CLSM analysis and arbitary fluorescence unit counting (AFU) for microbial analysis of *S. mutans*	Positive correlation between surface roughness and biofilm formation on ceramic CAD/CAM blocks and composite resins	Contreras-Guererro et al., 2020 [74]
Comparison of biofilm adhesion and formation on different smooth dental restorative materials with human enamel	(1) Ceram X, Dentsply-Sirona, Konstanz, Germany (2) IPS emax Press, Ivoclar Vivadent (3) Lava Plus, 3M ESPE (4) Vita Enamic, Vita Zahnfabric (5) metal alloy (CoCrMo) (6) human enamel (control group) POLISHING PROCEDURES Finished and polished according to the manufacturers’ instructions	(1) 3D optical profilometer for surface roughness evaluation (Sa height parameter) (2) SEM analysis and CFU/mL counting for microbiological analysis (3) Mass spectrometry for species identification	Biofilm maturation on specific restorative materials is influenced by surface properties and material composition Microbiological analysis showed that bacterial strains differed between the materials	Engel et al., 2020 [75]
Evaluation of surface roughness, biofilm formation, cytotoxicity and genotoxicity of three resin-based CAD/CAM materials	(1) Vita Enamic, Vita Zahnfabrik (2) Cerasmart, GC (3) Brilliant Crios, Coltene Whaledent AG POLISHING PROCEDURES All specimens were uniformly polished with silicone carbide paper discs up to 1200 grit, diamond grit polishing discs and a diamond polishing paste	(1) Non-contact optical profilometer + SEM for surface roughness evaluation (2) CFU/mL counting for microbial analysis of *S. mutans* and *Lactobacilli*	Brilliant Crios showed the highest biofilm formation values No statistically significant differences in surface roughness values between groups No statistically significant correlation between surface roughness and bacterial adhesion for all groups	Hassan et al., 2022 [76]
Comparison of physicomechanical properties and biofilm formation between resin-based hybrid materials	(1) Grandio Blocs, VOCO GmbH (2) Lava Untimate, 3M ESPE POLISHING PROCEDURES Materials were polished according to the manufacturer’s instructions	(1) Stylus profilometer for surface roughness evaluation (Ra height parameter) (2) SEM analysis and CFU/mL counting for microbial analysis of *S. mutans*	Grandio Blocs showed significantly lower roughness and bacterial adhesion when compared to Lava Ultimate Positive correlation between surface roughness and bacterial adherence for both resin-based CAD/CAM materials.	Mokhtar et al., 2022 [77]
Effect of different polishing techniques on surface properties and bacterial adhesion on resin-based CAD/CAM materials	(1) Vita Enamic, Vita Zahnfabrik (2) Lava Ultimate, 3M ESPE (3) Cerasmart, GC POLISHING PROCEDURES (1) Non-polished (control group) (2) Manually polished (3) Glazed	(1) Profilometer in contact mode for surface roughness evaluation (Ra height parameter) (2) Contact angle measurement for surface free energy evaluation (3) SEM/EDS analysis for elemental and topographical evaluation (4) CFU/mL counting and SEM analysis for microbial evaluation of *S. mutans*	Non-polished CAD/CAM controls showed the highest surface roughness values Non-polished CAD/CAM controls showed higher bacterial adhesion Positive correlation between polishing procedures, surface properties and bacterial adhesion	Ozarslan et al., 2022 [78]
Evaluation of surface roughness, surface wettability and biofilm formation on CAD/CAM and 3D-printed materials for permanent restorations	(1) Vita Enamic, Vita Zahnfabrik (2) Cerasmart, GC Corp. (3) Lava Unltimate, 3M ESPE (4) Varseo Smile Crown Plus, BEGO, Bremen, Germany (5) Saremco Print Crowntech, Saremco dental AG, Rebstein, Switzerland (6) Formlabs 3D Permanent Crown, Formlabs, Somerville, MA, USA POLISHING PROCEDURES Equally polished with 600–800-grit-sized silicon carbide discs and aluminum oxide-coated discs (coarse, medium, fine and extrafine discs)	(1) Profilometer in contact mode for surface roughness evaluation (Ra height parameter) (2) Contact angle measurement for surface wettability (3) CFU/mL counting and SEM analysis for microbiological analysis of *S. mutans* and *S. sanguis*	Different digital manufacturing techniques and material compositions affect surface roughness No statistically signifcant diference between the groups in contact angle values Microbial adhesion varies regarding the bacterial species tested No correlation between surface roughness and bacterial adhesion	Ozer et al., 2023 [79]

## Data Availability

Not applicable.
